# Long-term symptom control following resection of cervical lymphatic malformations: a case series

**DOI:** 10.1186/s40463-020-00415-8

**Published:** 2020-04-19

**Authors:** Ashoke Khanwalkar, Taher Valika, John Maddalozzo

**Affiliations:** 1 Department of Otolaryngology Head and Neck Surgery, Ann and Robert H. Lurie Children’s Hospital of Chicago, Northwestern Feinberg School of Medicine, 225 E Chicago Ave, Chicago, IL 60610 USA; 2 Department of Otolaryngology - Head and Neck Surgery, Northwestern University, 676 N St Clair, Suite 1325, Chicago, IL 60611 USA

**Keywords:** Lymphatic malformation, Lymphovascular malformation, Lymphangioma, Longterm outcomes, Viral infection, URI

## Abstract

**Background:**

Previous literature has reported on the incidence of short-term complications following resection of cervical lymphatic malformations (LMs) in children, however no research has yet investigated the long-term symptomatic course in these patients. This study aims to provide families and providers with an understanding of expectations for long-term symptom control, specifically in association with subsequent upper respiratory infections (URIs).

**Methods:**

A retrospective chart review produced a case series of patients who underwent resection of cervical LM at a pediatric tertiary care center between 2007 and 2016. Demographic data, disease characteristics, operative details, and postoperative care were evaluated. Telephone surveys were conducted to ascertain the course of postoperative symptoms at the surgical site.

**Results:**

Forty-three patients responded to the telephone survey. Thirty-seven (86.0%) had at least one postoperative surgical site symptom during subsequent URIs, with 28 (65.1%) reporting redness, 34 (79.1%) reporting swelling, and 18 (41.9%) reporting pain. Patients who experienced any of these symptoms universally indicated that they developed soon after the surgical resection, and over half reported that they improved over time. Postoperative seroma was associated with swelling during subsequent URIs (*p* = 0.04). Patients age 7 or were greater were more likely than those under 7 to report pain with URIs (*p* = 0.006). All 8 patients with drain placement for at least 2 days reported swelling during subsequent URIs. The incidence of the queried symptoms did not vary significantly based on sex, stage, histology, surgical subsite, or presence of residual disease.

**Conclusion:**

While preoperative symptoms associated with cervical LMs are also frequently encountered postoperatively, particularly in some patient subgroups, improvement over time should be expected.

**Level of evidence:**

4

## Introduction

Lymphatic malformations (LMs) represent one of the most common pediatric head and neck vascular malformations [1]. Although typically present at birth, they may only be identified later in childhood following infection or trauma, at which time they may become swollen, red, and painful. Lesions in the cervical region commonly have finger-like extensions that cross tissues planes making extirpation challenging. Intervention – including sclerotherapy and surgical excision – is typically pursued when the airway, speech, or feeding are affected, or to improve cosmesis.

Previous research by our group has elucidated the 30-day perioperative outcomes following resection of cervical LMs in the pediatric population, helping clinicians and families to make well-informed collaborative medical decisions [2]. Other groups have also identified the relationship between overall local control and complications associated with resection of LMs based on lesion and patient characteristics [3, 4]. Based on the senior author’s experience of over 30 years in the surgical management of LMs, it has been noted that many patients experience postoperative redness, swelling, or discomfort at the site of resection during viral upper respiratory infections (URIs) – similar to the preoperative symptomatology. Families often approach the surgeon postoperatively with concerns about this clinical finding, but little is known about the long-term course of these symptoms following surgical resection.

On review of the existing literature, the incidence, natural history, and associated time course of these particular symptoms during URIs after LM resection have not been recorded. Furthermore, the likelihood of these particular postoperative symptoms at the surgical site has not been assessed in relation to patient-specific or perioperative factors. Families and providers may benefit from an understanding of likely expectations following surgery. As one of the larger series of surgical cases identified in the literature, the present study aims to provide families and physicians with prognostic long-term surgical site symptom outcomes following resection of cervical LMs.

## Materials and methods

The data for this study were collected via a combination of retrospective chart review as previously described as well as telephone surveys to gather additional information from families [2]. This historical case series included patients under age 18 years who underwent surgery at Ann & Robert H. Lurie Children’s Hospital from June 1, 2007 to September 30, 2016. Long-term follow-up was defined as at least 1 year in order to allow for evolution in surgical site symptoms, and hence this cutoff date was selected to ensure that all patients met this criterion. The study was approved by the associated Institutional Review Board. The database query (Bio Integration Suite and Clarity) was performed by using relevant Current Procedure Terminology codes (38550, 38555), internal institutional procedure and billing codes, as well as procedure-specific keywords. The study focused specifically on cervical LMs. Charts were reviewed to confirm the diagnosis and the associated procedure.

Baseline and outcomes variables were collected, including patient demographics, disease characteristics (e.g. stage, histology), operative details (e.g. extent of surgery), post-operative care (e.g. length of drain placement), incidence of seroma, and presence of residual tumor postoperatively. Lesions were classified as microcystic (individual cysts < 2 cm in diameter), macrocystic (cysts > 2 cm in diameter), or mixed [5]. Lesions were staged according to the classification system devised by de Serres et al. [6], which remains in common use today (see Table 1).

**Table 1:** Staging: de Serres Classification of Lymphatic Malformations

Stage 1	Unilateral infrahyoid
Stage 2	Unilateral suprahyoid
Stage 3	Unilateral infrahyoid and suprahyoid
Stage 4	Bilateral suprahyoid
Stage 5	Bilateral infrahyoid and suprahyoid

The de Serres staging system for lymphatic malformations was described in 1995. It remains the standard means to classify the extent of the lesion. The system was designed to predict prognosis as well as outcomes and complications associated with surgical intervention

Telephone surveys were conducted with patients and their caregivers to obtain information on the long-term symptomatic course of the surgical site in relation to URIs, specifically regarding redness, swelling, and pain. Information was also gathered on the time course of these symptoms. A standardized questionnaire was developed based on the questions most frequently posed by patients and families during postoperative visits in the senior author’s experience (see Table 2). While not formally validated, the questionnaire was administered in a routine, systematic manner by a single surveyor to reduce variability in data acquisition and mitigate observer bias.

**Table 2:** Telephone Survey Questions

1.	Did you [your child] experience redness in the area of surgery during upper respiratory infections?
2.	Did you [your child] experience swelling in the area of surgery during upper respiratory infections?
3.	Did you [your child] experience pain or a change in sensation in the area of surgery during upper respiratory infections?
4.	If yes to any of the above, did the symptom occur immediately after surgery or did it develop at a future time?
5.	If yes to any of the above, has the symptom gotten better or worse since surgery?
6.	If yes to any of the above, has the symptom resolved, and if so after what period of time?

Patients were asked a routine set of questions that were analyzed for trends and associations in relation to their preoperative and perioperative factors

### Surgery

Surgical procedures were tailored to each individual patient’s extent of disease. For subset analyses, each case was characterized by the extent of surgery in regards to laterality as well as involvement of specific regions, including floor of mouth, submandibular gland, and parapharyngeal space. Patients undergoing parotidectomy as part of their surgery were excluded as the resection carries unique risks, such as sialocele and Frey syndrome, which may cloud the assessment of symptoms of interest. The year of surgery was dichotomized by the median reported surgical date into before 2012 versus 2012 and after. This analysis was performed to assess whether increased surgeon experience and optimization of technique impacted postoperative outcomes.

### Statistical analysis

Data were checked for normal distribution and outliers were removed. Records with incomplete information were excluded only from the corresponding analysis. Patient responses to survey questions were compared with patient and perioperative variables with a Fisher’s exact test. For this purpose, the stage of lesion was dichotomized into “lower stage lesions” (stages 1 and 2) and “higher stage lesions” (stages 3, 4, and 5). In regards to cyst type, microcystic and mixed lesions were grouped together and compared against purely macrocystic lesions. Age was dichotomized by the median and grouped into “younger” (less than 7) and “older” (7 or more). Length of drain placement was split into two or fewer days versus more than 2 days. Timing of surgery was split by the median date to be “earlier” (prior to 5/25/12) and “later” (on or after 5/25/12).

## Results

**Fig. 1:**
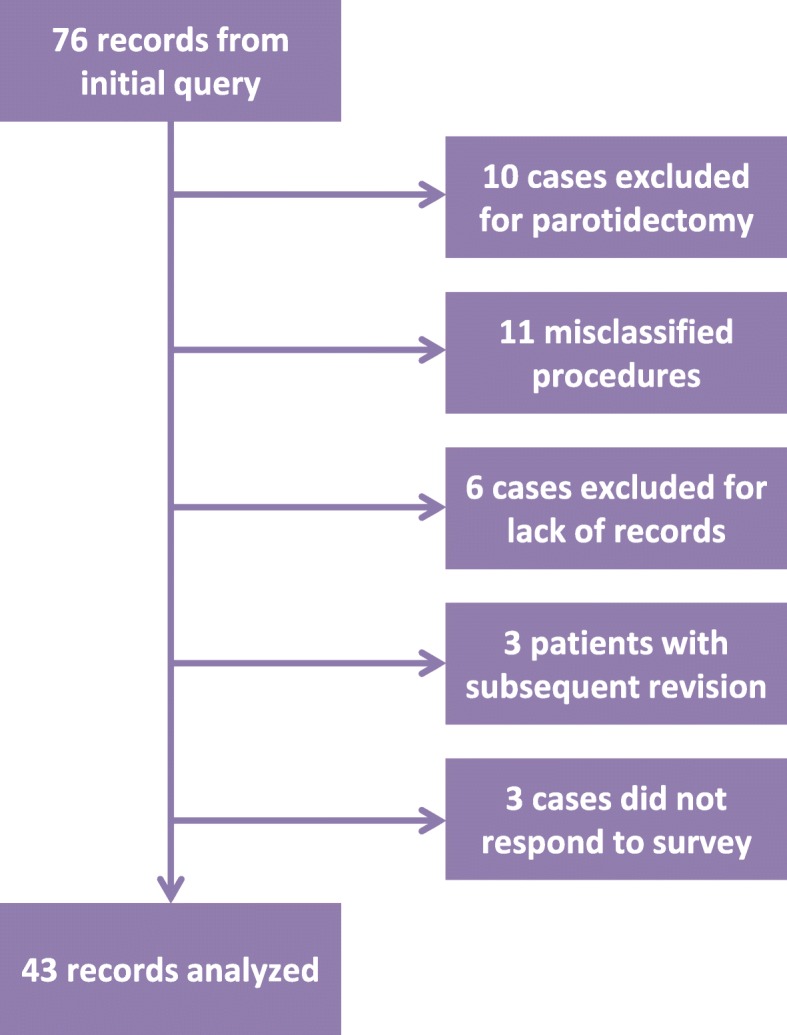
Case Series Acquisition. Flow chart of original case query and exclusions to produce the final set for analysis. The original query was based on CPT codes, internal institutional procedure and billing codes, as well as procedure-specific keywords. Patients having undergone parotidectomy were excluded due to the unique associated complications, and given that this population was addressed in another study. Misclassified procedures included entirely unrelated interventions, e.g. abscess drainage, which were inappropriately retrieved in the query. Three patients had revision procedures and only the most recent procedure was considered for long-term outcomes. Three patients did not respond to the telephone survey and so were not included for analysis

Seventy-six records were examined; of these, 27 cases were excluded due to associated parotidectomy, other procedures misclassified as excision of LM, or lack of associated records to analyze outcomes (see Fig. 1). Three of the remaining patients had multiple procedures performed; only the more recent procedure was considered in relation to the survey questions. Forty-three of the remaining 46 patients responded to the telephone survey and were included for analysis (see Table 3). Of this group, 7 cases were performed after prior excision alone, 1 after sclerotherapy alone, 1 after prior excision and sclerotherapy, and 1 after prior excision, sclerotherapy, and aspiration. Median age at time of surgery was 7 years (range 0–17), and the breakdown between sexes was nearly even. Median time to follow-up from surgery was 6.2 years (range 1.7–12.0). The included cases varied in stage, histology, and surgical subsite explored (see Table 4).

**Table 3:** Demographics

**Sex**	
Male	21 (48.8%)
Female	22 (51.2%)
**Stage**	
Stage 1	18 (41.9%)
Stage 2	11 (25.6%)
Stage 3	11 (25.6%)
Stage 4	0 (0.0%)
Stage 5	3 (7.0%)
**Cyst Type**	
Macrocystic	13 (30.2%)
Microcystic	8 (18.6%)
Mixed	22 (51.2%)
**Residual Cyst**	
Yes	16 (37.2%)
No	27 (62.8%)

Background demographic data on the final patient population included in the study. This information was used to evaluate predictive factors relating to symptoms at the resection site

**Table 4:** Surgical Details

**Laterality**	
Unilateral	40 (93.0%)
Bilateral	3 (7.0%)
**Extent**	
Submandibular gland excision	8 (18.6%)
Floor of mouth resection	14 (32.6%)
Parapharyngeal space	24 (55.8%)

Surgical details for patients in the series. Given that most patients had a stage 3 or lower lesion, it is expected that those associated surgeries would be unilateral. However, there was variety in the extent of surgery and anatomic areas in need of dissection. These data points were evaluated as predictors of the postoperative symptomatic course

Of the 43 patients who responded to the telephone survey, 37 (86.0%) had at least one of the queried postoperative surgical site issues in association with viral URIs. Twenty-eight patients (65.1%) reported redness, 34 (79.1%) reported swelling, and 18 (41.9%) reported pain or a change in sensation. Patients who experienced any of these symptoms universally indicated that they developed soon after the surgical resection. Of the 37 patients with surgical site issues, 11 (29.7%) had complete resolution within 1 year, while an additional 5 (13.5%) had resolution between 1 and 3 years. Four patients (10.8%) state the issues have entirely resolved but are uncertain of the timing. However, 15 patients (40.6%) were uncertain whether the issues have definitively resolved as of the time of interview. Two patients (5.4%) state that they still have symptomatic surgical site issues during URIs. The time to improvement did not vary notably between the symptoms assessed.

The incidence of each symptom did not vary significantly based on sex, stage, histology, surgical subsite explored, year of surgery, or presence of residual disease (see Tables 5 and 6). All patients who had a postoperative seroma experienced swelling at the surgical site during URIs, which was a significant difference from those without seroma (13/13 versus 21/30, *p* = 0.04). Older patients were more likely than younger patients to report pain at the site during URIs (13/20 versus 5/23, *p* = 0.006). While the impact of duration of drain placement did not reach statistical significance, all 8 patients with a drain for more than 2 days reported swelling at the surgical site during URIs, while only 26 of the 35 patients who had a drain for 2 or fewer days were affected.

**Table 5:** Symptom Incidence by Patient or Perioperative Risk Factor

Risk Factor	Redness	Swelling	Pain
Residual disease	16/27	21/27	14/27
No residual disease	12/16	13/16	4/16
	*p* = 0.3	*p* = 1	*p* = 0.1
Lower stage (1 to 2)	18/29	21/29	12/29
Higher stage (3 to 5)	10/14	13/14	6/14
	*p* = 0.7	*p* = 0.2	*p* = 1
Purely macrocystic	8/13	10/13	6/13
Microcystic and mixed	20/30	24/30	12/30
	*p* = 0.7	*p* = 1	*p* = 0.7
No seroma	18/30	21/30	12/29
Seroma	10/13	13/13	6/14
	*p* = 0.5	*p* = 0.04	*p* = 1
Age < 7	15/23	19/23	5/23
Age ≥ 7	13/20	15/20	13/20
	*p* = 1	*p* = 0.7	*p* = 0.006
Male	13/21	16/21	8/21
Female	15/22	18/22	10/22
	*p* = 0.8	*p* = 0.7	*p* = 0.8
Surgery before 2012	15/22	16/22	9/22
Surgery 2012 and after	13/21	18/21	9/21
	*p* = 0.8	*p* = 0.5	*p* = 1
Drain ≤2 days	23/35	26/35	15/35
Drain > 2 days	5/8	8/8	3/8
	*p* = 1	*p* = 0.2	*p* = 0.4

Patient and perioperative risk factors and association with postoperative surgical site symptoms during viral URIs. Fisher’s exact test used for comparisons

**Table 6:** Symptom Incidence by Surgical Subsite Explored

Surgical Subsite	Redness	Swelling	Pain
No submandibular gland excision	21/35	27/35	14/35
Submandibular gland excision	7/8	7/8	4/8
	*p* = 0.2	*p* = 1	*p* = 0.7
No floor of mouth excision	17/29	22/29	13/29
Floor of mouth excision	11/14	12/14	5/14
	*p* = 0.3	*p* = 0.7	*p* = 0.7
No parapharyngeal space exploration	13/19	14/19	9/19
Parapharyngeal space exploration	15/24	20/24	9/24
	*p* = 0.8	*p* = 0.5	*p* = 0.5

Common surgical subsites explored with associated postoperative symptoms. There was no significant association between extent of surgery involving these subsites and symptom outcomes. Fisher’s exact test used for comparisons

## Discussion

LMs remain one of the most common head and neck vascular lesions in the pediatric population, and the literature has recently expanded in regards to postoperative complications and recurrence rates. Several studies have examined short-term outcomes following resection of LMs in the head and neck – our group previously published the largest series at that time on 30-day postsurgical complications [2], and other groups have also contributed to this literature [3–5, 7–10]. Wang et al. report an impressive case series of 128 children with LMs followed postoperatively for a median of 3 months (range 5 days to 8 years and 8 months) and evaluated for control rates, complications, and recurrence rates associated with patient and perioperative factors [3]. Lei et al. similarly reported on complications and recurrence rates in a series of 117 patients, 89 of whom were followed for an average of 3.7 years (range 6 months to 11 years) [4]. Some of the complications explored included facial nerve injury, infection, hematoma, seroma, external deformity, and salivary fistula. Benazzou et al. highlighted the long-term outcomes after a mean 15 months follow-up and the challenges associated with removal of massive LMs, but did not provide detailed prognostic information based on patient or perioperative factors [11]. Lerat et al. followed 23 patients over a mean of 27.65 months, while Ma et al. followed 68 patients over mean 27.8 months (range 3 to 60 months), and tracked outcomes such as recurrence and identified complications such as infection, edema, and nerve weakness [12, 13]. The long-term follow-up duration of the present study compares favorably with the existing literature, but explores a specific set of concerns for patients and families that has not been directly addressed to our knowledge.

Swelling, redness, and pain at the site of the lesion are common findings during URIs in patients who present with LMs; however, similar issues may persist after intervention. No study appears to have investigated the incidence of these symptoms postoperatively in relation to patient and perioperative factors. Families and providers may be uncertain whether this is normal and question whether to expect improvement. Establishing expectations based on data regarding the normal postoperative course can help parents and providers to understand the typical course following surgical resection of cervical LMs.

This study aims to analyze long-term surgical site issues following excision of cervical LMs in the pediatric population at a tertiary academic medical center. Telephone surveys were used to contact patients and caregivers and assess the actual incidence of noted issues including redness, swelling, and pain during URIs. Patients very commonly reported at least one of these issues at the surgical site developing soon after surgery, with over 86% indicating at least one of the above symptoms in association with URIs. However, despite the near ubiquity with which they occur, over half report definitive improvement of any existing symptoms over time. While many patients were uncertain about complete resolution of symptoms, only 2 of the 43 patients definitively reported that they still experienced these issues at time of follow-up. The existing literature has described the natural history of LMs with a spontaneous resolution rate from 12.5 to 45.5%, but these patients typically have low stage purely macrocystic lesions, lending some support to intervention for at least a subset of LMs [14–16].

While higher lesion stage and microcystic histology were associated with an increased rate of 30-day postoperative complications in prior studies including our own [2, 17–20], there did not appear to be an association with long-term incidence of redness, swelling, or pain. Neither the surgical subsite nor the presence of residual disease seemed to play a role either. Other cited studies have found higher rates of complications in patients with lesion higher stage and more anatomically extensive disease [3, 4], but the particular symptoms of interest in this study did not vary along these parameters, although this may perhaps be due to a lack of power. The year of surgery was dichotomized by median surgical date and investigated to evaluate whether there was a change in surgical technique over time that led to different results, but this too did not influence the incidence of these findings.

However, some patient and perioperative factors did influence the likelihood of reported symptoms. Patients age 7 or greater were more likely to report discomfort at the surgical site during URIs. It remains unclear whether this finding is simply due to their greater ability to express their discomfort to parents or physicians compared with younger patients. Every patient who had a postoperative seroma also reported surgical site swelling during subsequent URIs, which could possibly be attributed to residual lesion, or possibly to a large resection cavity that offers a potential space to fill. Further, every patient who required a drain for more than 2 days also reported these symptoms over the weeks to months after surgery. There was not a relationship between longer drain placement and seroma formation, which appears to suggest these are independent risk factors.

This study has some notable limitations. The context is an academic medical center and so may represent findings associated with a high volume setting. Nevertheless, despite representing a relatively large series for this particular pathology, the small number of total cases limits conclusions that can be drawn. The study relies on telephone surveys of patients and parents regarding experiences from the distant past, and so recall bias is inherent. While observer bias also plays a role through the administration of a non-validated questionnaire, variability was mitigated as much as possible through use of a single surveyor relying on preset questions. Finally, given that the study is not a randomized controlled trial, it can only demonstrate association, not causation.

## Conclusion

While relatively few studies have investigated short-term outcomes following surgical intervention for pediatric LMs of the neck, and fewer still have looked at long-term symptom control, none have evaluated the incidence of persistent postoperative URI-associated symptoms at the surgical site in combination with prognostic factors. This case series is the only study identified to evaluate swelling, redness, and pain at the surgical site during URIs following resection of a cervical LM. While the occurrence of these symptoms is very common, particularly in some subgroups, the issues tend to improve with time. The findings presented in this study may provide useful information to clinicians and families for counseling on expectations following resection of these lesions. Further studies should be designed to investigate the relationship between drain duration, seromas, and incidence of these symptoms, and to describe outcomes in other healthcare delivery settings.

## Data Availability

The datasets generated and analysed during the current study are not publicly available to privacy concerns, but are available from the corresponding author on reasonable request.

## Abbreviations

LM: Lymphatic malformation
URI: Upper respiratory infection
